# Nail Polishes: A Review on Composition, Presence of Toxic Components, and Inadequate Labeling

**DOI:** 10.1155/drp/6330337

**Published:** 2025-03-06

**Authors:** Aislana Cole de Paula, Fabrício Uliana, Eloi Alves da Silva Filho, Priscilla Paiva Luz

**Affiliations:** Department of Chemistry, Federal University of Espírito Santo, Vitoria, Espírito Santo, Brazil

**Keywords:** cosmetics, formulation, nail polish, polymers, toxic components

## Abstract

Nail polishes were developed in 1920, and since 1940, it has been known that these cosmetics contain toxic and sensitizing components. Over the years, nail polishes have undergone several changes in their formulation to avoid this problem, but new components have also been considered toxic and allergenic. The growing demand for gel nails has also been highlighted in cases of allergy to (meth)acrylates, and the biggest concern that was previously related to the presence of toluene sulfonamide-formaldehyde resin (TSFR) in traditional nail polish formulations is now also part of (meth)acrylate-based cosmetics. The beautification caused by nail polish is the main factor behind its constant use throughout the world, but studies have demonstrated its use for other purposes, such as treating fungal diseases, sun protection factor in cancer patients, and as a possible ally in forensic area. This review brings the beginning of the discovery of nail polish and its trajectory to the present day, including its effects on health and its inadequate labeling. Therefore, it is extremely important that legislation monitors the composition of nail cosmetics and that new formulations are studied to make them safe for health and the environment.

## 1. Introduction

The practice of coloring and adorning nails dates to antiquity, and the earliest evidence dates to 6000 BC, when archaeological excavations unearthed period nail polish, as well as face powder and eye makeup. In ancient China and Egypt, henna leaves and clay were used to dye nails a reddish-brown color for many years. There are also records of a mixture used by the Chinese made of gum arabic, beeswax, egg whites, gelatin, and flower petals to be used on their nails, around 3000 BC. In China and Egypt, red and dark tones indicated social status, being reserved only for royalty, while the lower class had light tones. Still in Egypt, leaders such as Queen Nefertiti (between 1375 and 1350 BC) and Cleopatra (between 69 and 30 BC) were known for keeping their nails painted. While Nefertiti was known for her ruby-red nails, Cleopatra preferred rust-colored tones [1–3].

During the 5th century BC, a light shade was obtained through a mixture of yellow flower petals, pollen, and potassium salt [3]. During a period of the Roman Empire (until 476 AD), the custom of keeping nails polished was acquired and the act of painting them ended up being left behind. During the Middle Ages (10–15th century), the modesty of the time did not allow strong tones on the nails, and it was preferable to keep them short and natural, only hydrated with creams and oils [4]. Long years passed and nail polishes (nail lacquer, enamel, or varnish) as we know them today were developed in the 1920s, as a result of the progress made in the automotive industries after the discovery of nitrocellulose (NC) as a film-forming polymer. From then on, the first colored nail polish was developed in 1930, when Charles Revson incorporated pigments into the transparent nail polish, and in 1932, the first nail polish company was founded, Revlon, a renowned company that remains to this day. Although NC is no longer used in automotive paints, it is still the most used primary film-forming polymer in nail polish formulations [2, 5, 6].

In nail polish formulation, NC is not used alone as a film former, as the film formed is brittle and has low adhesion to the nails. Therefore, it is necessary to use other components to obtain the best properties. The biggest problem with nail polish is the presence of components considered toxic and/or allergenic. The need to use additives such as thermoplastic resins and plasticizers has led industries to use toxic components such as toluene sulfonamide-formaldehyde resin (TSFR) used as a thermoplastic resin and dibutyl phthalate (DBP) used as a plasticizer [2, 7, 8].

## 2. Methods

A bibliographical search was carried out in the Web of Science and Scopus databases Web of Science and Scopus. The same keyword search terms and Boolean operators were used for each search engine: “nail polish” OR “nail lacquer” OR “nail varnish” OR “gel nail polish” OR “nail cosmetics” OR “nitrocellulose.” Publications related to water-based nail polish, teeth, pulse oximetry, and electrochemical sensors were not considered. Full articles, reviews, and case reports were selected to assess their relevance.

## 3. Discussion

### 3.1. Nail Polish Composition

Nail polishes are basically made up of film-forming polymers (∼15%), thermoplastic resins (∼7%), plasticizers (∼7%), solvents (∼70%), pigments (< 1), and suspending agents (∼1%) [2, 9–12].  Table 1 shows the main components of nail polishes used over the years.

#### 3.1.1. Nitrocellulose (NC)

NC is obtained from cellulose in an esterification reaction with a sulfonic acid mixture between sulfuric (H_2_SO_4_) and nitric (HNO_3_) acids. Each glucose molecule has three hydroxyl groups available for esterification reactions. Substituting one, two, or three of the hydrogen atoms in the OH groups with NO_2_ groups results in mono-, di-, or trisubstituted cellulose, respectively. This leads to polymers with varying degrees of substitution. The degree of substitution (DS) is defined as the average number of hydroxyl groups replaced in each glucose unit. It is a relevant property because the physical properties of the polymer are affected by the amount of NO_2_ groups in its structure [13, 14]. The nitrogen content or DS is one of the main points to be considered when choosing the NC, as NC with a nitrogen content lower than 12.5% (between 10.8% and 12.3%) is widely used as a raw material in printing inks, lacquers, varnishes, nail polishes, and adhesives due to the ease of being solubilized in organic solvents, such as acetates and ketones. On the other hand, solubility decreases with increasing nitrogen content and highly nitrated NC (nitrogen content above 12.5%) is used in the manufacture of propellants and explosive materials, being called explosive grade NC [15–17].

To formulate nail polish, the NC is dissolved in a mixture of organic solvents whose function is to keep the nail polish homogeneous in the bottle, adjust the viscosity, and leveling of the film, that is, to assist in the uniform distribution of the nail polish, in addition to regulating the drying speed. A mixture of solvents with different boiling points (BPs) is used to ensure the formation of a more durable and bubble-free film. Solvents used in nail polishes are classified according to their BPs, such as quick-drying solvents (or light solvent), intermediate (or medium solvent), and slow (or heavy solvent) and they can also be classified as active solvents (or true solvents), co-solvents, and non-solvents. Solvents called active are true solvents, as they are miscible with NC and provide clear solutions. Co-solvents are latent solvents, as they have limited action on the solubility of NC, but they act in synergy with active solvents and assist in their solubility. The most used example in nail polish formulations is isopropyl alcohol, with a BP of 82.5°C. Non-solvents are added to reduce costs and aid viscosity, such as toluene [2, 5, 18, 19].

The initial replacement of components such as TSRF and DBF was a challenge for the cosmetic industry as both worked very well with NC. The polymer is unlikely to be replaced as a film former in nail polishes [5, 20], as it has been considered safe according to the Cosmetic Ingredient Review Expert Panel [8, 21].

In 1994, NC was reported as a potential sensitizer, and between 1977 and 1983, only one case of allergy to the polymer was reported among 13,126 patients tested [22]. In 2015, oral toxicity studies in mammals showed no toxicity effect in relation to NC [23]. Even with so many changes in the composition of nail polishes, NC remains present in most formulations [5, 10, 11, 20], and according to Fiume et al. [14], data obtained by the FDA in 2013 showed that 568 nail product formulations, including bases and nail polishes, were analyzed and 516 showed significant amounts of NC, which is expected, as it is used as a film former in nail polishes. In 410 formulations, the amount of NC was in the range of 13%–22% of the total composition. In his research, the author also claimed that NC did not produce toxic, carcinogenic, or mutagenic effects when tested on animals, and any type of irritation or sensitization was observed when tested on humans, concluding its safety in cosmetic products. Couteau, Paparis, and Coiffard [10] analyzed 65 nail polish brands, and all were made up of NC, and in 2018, the authors carried out another study with 50 nail polishes and only one did not contain NC, as it was an aqueous gel [11]. Young et al. [24] examined 55 nail polishes considered between 3-Free and 13-Free, and only one sample of watery nail polish did not contain NC as an ingredient. In their study, Jimenez et al. [12] identified NC in 12 nail polish samples analyzed from three different brands. Due to its low cost, raw material availability, and renewable sources (cotton and wood), it is unlikely that NC will be replaced as the primary film-forming polymer in nail polishes [5].

#### 3.1.2. Toxic Components Present in Nail Polish: “Toxic Trio”

Nail cosmetics are known to cause allergic contact dermatitis (ACD), which can present in different ways, from specific nail problems to other symptoms that resemble systemic diseases [25–27]. Sainio et al. [7] already reported that nail polish contained allergenic components more than 50 years ago. Initially, these components were called “toxic trio,” namely, TSFR resin, DBP plasticizer, and toluene.

##### 3.1.2.1. Toluene Sulfonamide-Formaldehyde Resin (TSFR)

TSFR resin is formed from a condensation reaction between p-toluene sulfonamide and formaldehyde and was introduced into nail polish in the late 1930s, being the thermoplastic resin of choice for many years. In 1940, the first cases of ACD related to the use of TSFR were identified, and for a long time, it was the most investigated allergen, being among the main standard dermatology tests [7, 28–30]. Although it is an effective secondary film former for NC, concerns about residual formaldehyde have led to a decline. TSFR is used since the 1990s, as formaldehyde was classified as a carcinogen [31].

In 2015, the European Scientific Committee on Consumer Safety (SCCS) established the maximum use of 2.2% formaldehyde in nail hardeners, which was previously 5%, with the aim of alleviating cases of allergies related to the component [8]. In nail strengthening bases, formaldehyde is the active ingredient, and for sensitized individuals, a concentration of formaldehyde as low as 0.006% can trigger an ACD [25, 26].

A study based on 1405 medical records of patients treated between 2004 and 2017 confirmed the diagnosis of ACD related to TSFR in 29.7% of patients [32]. In this sense, other resins began to be explored to replace TSFR, such as polyester resins (glycerophthalic polyester resin, phthalic polyester resin, and polyester-saturated hydroxylated resin), epoxy resins (4-methylbenzene sulfonamide-epoxy resin), resins based on acrylates and methacrylates, cellulose acetate butyrate, among others, but over time, these resins were also considered highly sensitizing [26, 29, 33].

In 2022, the formaldehyde was banned in cosmetic products, with the exception of preservatives that gradually release formaldehyde in the formulations. However, it is mandatory that it be described on the labels when the concentration of formaldehyde in the finished product is higher than 0.05%. The SCCS states that the 0.05% limit may still sensitize some patients, and that to increase the protection of these consumers, the limit should be reduced to 0.001% [34].

##### 3.1.2.2. Dibutylphthalate (DBP)

In the early 2000s, DBP was introduced into formulations as a plasticizer due to its excellent interaction with NC. DBP was the plasticizer most used by the nail polish industry, but animal studies identified the plasticizer as being toxic to the reproductive and developmental systems [24, 35]. In 2001, the National Report on Human Exposure to Environmental Chemicals noted high levels of phthalates excreted by women of reproductive age, but phthalates present in cosmetics were not considered harmful to health due to the lack of a scientific basis to support such speculations. Thus, the reproductive risks related to exposure to phthalates were considered minimal by the Food and Drug Administration (FDA) [36].

Chronic animal studies have confirmed that phthalate esters cause developmental and reproductive toxicities, and the FDA has since monitored their use in cosmetics. However, available data on the effects on human health related to exposure to phthalates are scarce and divide opinions: While one group considers DBP to be safe in the quantities used, one group advocates its elimination as it poses a danger to human health. In view of the above, there was concern about eliminating phthalates from cosmetic products, and due to their potential level of toxicity on reproductive health, possible carcinogenicity, congenital defects, and changes in thyroid function, the use of phthalate esters was banned by the European Union in 2004 and, later, by some US states [7, 24, 31, 37]. A 2002 survey found phthalate esters in 100% of the nail polishes analyzed [38], and according to its last cosmetics survey in 2010, the FDA reported that DBP is rarely used, but the few subsequent studies on phthalates in nail polish confirm that the component is still present in some brands of nail polish [24, 37, 39, 40]. Hubinger [38] investigated by high-performance liquid chromatography (HPLC) whether the nail polishes that were positive for phthalates in the research carried out in 2002 had been reformulated and identified that 11 of the 24 nail polishes analyzed still contained at least one phthalate ester, and the highest concentrations were for DBP, ranging from 123 μg/g to 62,607 μg/g.

To replace DBP, industries started using triphenyl phosphate (TPHP); however, studies suggest that TPHP is an endocrine disruptor that can adversely affect thyroid function [24, 37, 40, 41]. Exposure to TPHP is also related to reproductive toxicity, decreasing male fertility. Delayed puberty was observed in male and female rats, and it was also reported that TPHP crosses the blood–brain barrier in mice, suggesting neurotoxicity [42]. Data on its toxicity in humans are scarce, but the plasticizer has been included in The Endocrine Disruption Exchange's current list of endocrine disruptors, as it has been shown to be a potential risk to human health [43]. The plasticizer on the rise, which began to be used in formulations to replace those considered toxic, is acetyl tributyl citrate (ATBC), considered safe and approved by the FDA [10, 20, 44].

##### 3.1.2.3. Toluene

Toluene, considered the third component of the “toxic trio,” was widely used in nail polish as a non-solvent to aid the evaporation rate of the formulation and stabilize viscosity, but its addition in nail polishes has also shown a decline since the 1990s, when the California Air Resources Board imposed restrictions on its use because it causes adverse effects on the central nervous system (CNS), cardiovascular and respiratory systems, and reproductive toxicity, in addition to being considered carcinogenic and teratogenic [6, 8, 35].

#### 3.1.3. Nail Polishes “Toxic-Free”

Due to the problems presented by the components initially considered toxic, TSFR, DBP, and toluene, many manufacturers invested in new formulations and called themselves free from the considered toxic trio. This “new generation” of nail polishes arrived on the shelves in 2006 with new labels, indicating being hypoallergenic, leading to the emergence of “3-free” nail polishes, as they are free of TSFR, DBP, and formaldehyde [24, 31]. Formulations called 3-free quickly gained fame, but over the years, new components present in these formulations were also considered toxic and/or sensitizing, such as camphor, the first plasticizer used in nail polishes, epoxy resin, shellac, adipic acid-neopentyl glycol-trimellitic anhydride copolymer (AA), phthalic anhydride-trimellitic anhydride-glycol copolymer (PA), and benzophenones [24, 33, 45]. Some nail polish brands choose to use UV filters in their formulations, with benzophenone-1 and benzophenone-3 being widely used. However, it is known that benzophenones are highly sensitizing, being an important factor to be taken into consideration when choosing nail polish [10].

The replacement of the “toxic trio” raises the question that one toxic chemical has been replaced by another and shows that there is a long period between the identification of allergens and their restriction. Currently, there are some variations for the labels of nail polishes considered “toxic-free,” ranging from 3-free to 16-free. For formulations labeled above 3-free, other components are absent, such as camphor, other phthalates, acetone, ethyl tosylamide, parabens, xylene, TPHP, epoxy resin, ingredients of animal origin, bisphenol A, gluten, soy, fat, etc. [20, 24, 31, 46]. The truth is that this labeling often ends up being just a marketing ploy, as happens with nail polishes described as free from gluten, soy, and fat, since these components are not part of the nail polish formulation. Lead consumers who are new to the subject believe that they are purchasing a safe product. Another issue that needs to be reviewed is the lack of conformity between nail polish labels; for example, a label called 5-free from a certain brand does not always refer to the exclusion of the same components as a 5-free label from another brand and so onwards [20, 24].

#### 3.1.4. Are the Nail Polishes Really “Toxic-Free”?

The lack of a pre-commercialization authorization procedure for nail polishes by regulatory bodies directly implies their safety. In 2012, the California Environmental Protection Agency analyzed 23 nail polishes, 7 of which were called 3-free, but only 2 were free of TSFR, DBP, and toluene, with 5 having high concentrations of DBP and toluene. Of the 12 nail polishes that claimed to be toluene-free, the reagent was present in 10 samples and some concentrations were considered high (6.9%, 7.30%, 13%, and even 17.7%). Regarding DBP, higher concentrations were found in nail polishes labeled 3-free or more than in common nail polishes, ranging from 6.2% to 8.8%, compared to 1.4%–4.2% in common nail polishes. Other plasticizers considered problematic were also present in the nail polishes analyzed: camphor in 11 samples and TPHP in 5 samples [24, 39].

Mendelsohn et al. [47] investigated TPHP levels in 10 nail polishes, and the component was quantified in eight samples, of which the plasticizer was not even included in the list of ingredients in two of them. The result leads to the conclusion that the percentage of nail polishes containing TPHP is much higher, since the label does not list all the ingredients. In 2016, the presence of toluene was investigated in 29 nail polishes, being detected in 26 samples [48]. In 2017, a study carried out in Brazil examined 25 brands of nail polish called hypoallergenic, both national and foreign. Fifteen brands had at least one component considered allergenic, such as epoxy resin, TSFR, formaldehyde, and toluene in 13, 11, 2, and 1 samples, respectively [28].

In another study involving hypoallergenic nail polishes, 40 samples of 12 different brands of nail polish were analyzed and TPHP was found in 25 samples, with TPHP not appearing on the label in 12 of them. DEP was identified in three samples considered phthalate-free [24]. Tokumura et al. [40] examined the concentration of plasticizers present in 45 nail polishes purchased between 2013 and 2018 and determined the presence of ATBC, camphor, TPHP, and DBP in 35, 3, 3, and 1 samples, respectively. Voller et al. [31] analyzed 29 nail polishes that claimed to be formaldehyde-free (3-free or more), but the component was found in four samples. Lim et al. [43] investigated the presence of phthalates in Korean cosmetics, and DBP was found in 27 of the 31 nail polish samples analyzed, reaching a concentration of 46.3 mg/g.

In the study carried out by Heaton et al. [49], formaldehyde was detected in all samples except one, the FingerPaints Framed in Red, and in five samples, it was at concentrations above the recommended exposure limit. A study carried out in Brazil by De Menezes, Gomes, and Machado [50] analyzed 57 nail polish samples from national and international brands, including 3-free, 4-free, 5-free, and toxic-free samples; TSFR resin was identified in 52 samples, as was formaldehyde. Phthalates were identified in 23 nail polishes, camphor in 2 nail polishes, xylene in 8 nail polishes, benzophenone-1 in 9 nail polishes, and benzophenone-3 in 3 nail polishes. In two samples described as 3-free, toluene was identified, while in one of them, there was also DBP and formaldehyde. In two samples described as 4-free, toluene and TSFR were identified. Another sample described as 5-free presented DBP in its composition, just as samples considered toxic-free presented toluene, DBP, and TSFR.

Dural [51] investigated the presence of five phthalates in different cosmetics by HPLC, including 18 nail polish samples. Dimethyl phthalate (DMP) was identified in 15 samples, DBP in 9 samples, benzyl butyl phthalate (BBP) in 10 samples, DEP in 16 samples, and di(2-ethylhexyl) phthalate) (DEHP) in 7 nail polish samples. According to Estill et al. [41], in 2020, TPHP was identified in more than 700 nail polishes. While in their study, out of 11 nail polishes analyzed, 8 contained TPHP. In 2021, TPHP was identified in more than 1100 nail cosmetics [42]. Tang et al. [37] confirmed the presence of some harmful components, such as DEP, DBP, and TPHP in 27% of the nail polish brands analyzed. The results presented by the authors above show the discrepancy between the list of ingredients reported on the labels and the actual composition of nail polish formulations. In the end, the consumer suffers due to inadequate nail polish labeling.

#### 3.1.5. Main Harmful Components in Nail Polishes

As reported in items 3.1.3 and 3.1.4, replacement with new components does not always mean that the cosmetic has become 100% free of harmful components. Listed below are the main components considered toxic and allergenic or sensitizing, present in nail polishes:

• Allergenic/sensitizing components: TSFR, formaldehyde, camphor, epoxy resins, polyester resins, resins based on acrylates and methacrylates, cellulose acetate butyrate, phthalic anhydride-trimellitic anhydride-glycol copolymer (PA), adipic acid–neopentyl glycol–trimellitic anhydride copolymer (AA), benzophenones, and Shellac [26, 28, 31, 33].
• Toxic components: toluene, formaldehyde, phthalates, and TPHP [24, 31, 37, 52].

#### 3.1.6. Legislation

The International Cooperation on Cosmetics Regulation (ICCR) was created with the aim of aligning cosmetic regulations and increasing the level of consumer protection, comprising the United States, Japan, Brazil, the European Union, and Canada. The European standard on cosmetic products is considered the most complete and used as a reference for other countries [53]. The Commission of the European Union established the Cosmetics Regulation (CE) No. 1223/2009, where nail polishes must be considered safe for the consumer before being sold and the responsibility for risk assessment lies on the industry that produces the cosmetic, whose list of components must appear on the label in descending order of concentration (EU, 2009) [54]. Companies need to collect information about adverse effects and other complaints about their products through a cosmetovigilance system [53].

In the United States, the FDA established the Federal Food, Drug, and Cosmetic Act, and although there is a restriction on components that have established adverse effects, it does not require that products be tested for safety or approved before entering the market, except for dyes and pigments. It is up to the industry to self-regulate [8, 24, 36, 53]. Although the FDA is responsible for regulating cosmetics, it does not have the legal authority to remove cosmetics from the market that pose a threat to consumer health. Regarding Japan, the country also does not require pre-approval of cosmetics before they are sold, and manufacturers are responsible for ensuring the safety of their products. However, each cosmetic product must be notified before being manufactured or imported, enabling its identification [53].

In Brazil, the federal agency that regulates the industrial use of chemical substances is ANVISA (*Agência Nacional de Vigilância Sanitária*), which establishes the maximum quantity that can be used in formulations through resolutions, leaving it up to the industries to use these components in the determined quantity or replace them with others that are less harmful. In relation to the “toxic trio,” ANVISA allows a maximum concentration in the final product of up to 25% for toluene, up to 15% for DBP, and a maximum of 5% for formaldehyde, present in TSFR and nail hardeners [55–57]. In relation to Canada, cosmetic products also do not require pre-approval before their commercialization, but notification is required to allow tracking in the event of any complications. Notification of a cosmetic product does not affirm its approval by Health Canada, and since 2011, the cosmetic program called Canada Consumer Product Safety (CCPSA) has come into effect, which oversees the regulation of these products and states: “*no one shall sell any cosmetic that contains any substance that could cause harm to the user's health when the cosmetic is used*” [53].

### 3.2. Nail Cosmetics Based on (Meth)Acrylates

In addition to the traditional nail polishes reported so far, there are also nail cosmetics that are essentially based on (meth)acrylates. (Meth)acrylate polymers are formed from the polymerization of esters of acrylic (acrylates) and methacrylic acids (methacrylates). Polymerization occurs spontaneously or induced by ultraviolet (UV) light [58–61]. Among them, gel nails, acrylic nails, and gel or long-lasting nail polishes stand out, all of them very common among consumers. Both gel nails and gel polishes require UV light for polymerization, while acrylic nails are formed from a mixture of powdered polymer mixed with liquid monomer and do not require UV light, as polymerization occurs spontaneously, in the presence of catalyst molecules [26, 27, 62, 63]. Both gel nails and acrylic nails are generally used to lengthen natural nails, whereas gel nail polish does not have the ability to lengthen.

These cosmetics have gained a lot of popularity due to their durability compared to traditional nail polishes, as they are highly resistant to cracking and various solvents. While gel nail polishes last ∼15 days, gel and acrylic nails can last for 30 days or more. It is well documented that these components are potent sensitizers [26, 27, 64, 65]. (Meth)acrylates have been used in nail cosmetics since the 1950s, and allergies related to these cosmetics were first reported in 1956 [58, 59, 66, 67].

Methyl methacrylate (MMA) was one of the most used monomers, but due to serious cases of contact dermatitis, its use was banned in 32 US states and in Europe. Since 1970, the FDA has recommended to not use MMA in its pure form, as it is known as a highly sensitizing monomer, responsible for serious allergic reactions, including neuropathy and permanent nail loss [8, 64]. Other (meth)acrylate monomers were chosen because they were considered safer, but sensitization related to them has also been widely documented, including 2-hydroxyethyl methacrylate (HEMA), 2-hydroxypropyl methacrylate (HPMA), ethyleneglycol dimethacrylate (EGDMA), triethyleneglycol diacrylate (TREGDA), tetrahydrofurfuryl methacrylate (THFMA), and 2-hydroxyethyl acrylate (HEA) [26, 58, 62, 65].

Studies have indicated HEMA as the methacrylate with the most reaction in ACD tests, being a good screening agent for contact allergies to other (meth)acrylates [61, 62, 68, 69]. After being applied, the monomer is cured into a polymeric structure using UV light. In monomeric form, HEMA is considered a potent allergen, but in polymeric form, it has been considered safe. However, the polymerization process of (meth)acrylate monomers often occurs incompletely, leaving behind residual monomers responsible for the induction of ACD [61–63]. In 2007, HEMA was added to the North American Contact Dermatitis Group (NACDG) screening tray. In 2019, it was included by the European Contact Dermatitis Society in the European reference series for routine tests, and in 2020, it was restricted to professional use only, with the words “may cause an allergic reaction” on the label [61, 64, 68, 69]. In 2015, an investigation was carried out on the labels of 91 gel nail polishes, and HEMA was present in 46 nail polishes. In 2023, an analysis of the labels of 394 nail cosmetics based on (meth)acrylates from different countries (Netherlands, Poland, China, USA) identified 18 acrylates and 29 methacrylates, with HEMA being the most identified component, present in 229 products [69]. Suuronen et al. [70] analyzed 37 products in this category by gas chromatography–mass spectrometry (GC–MS) and identified (meth)acrylates in 32 samples, with HEMA being present in 20, but only 12 were described on the label.

A worrying issue regarding these cosmetics is the availability of kits for home use. There is no oversight for these kits, and consumers do not have any type of training to apply gel nails at home [25–27]. A 2016 study identified 65 consumers with reactions related to at-home gel polish kits [66]. The increase in cases of ACD related to nail cosmetics based on (meth)acrylates has been a worrying issue, as the sensitization caused by these cosmetics puts future medical interventions at risk and can generate a cross-reaction in patients, as the (met)acrylates are present in several products from different areas, including medical and dental, such as surgical glue, hearing aids, glucose sensors, bandages, orthopedic prostheses, contact lenses, dental prostheses, dentures, fillings, among others. It is advisable that, once sensitized to at least one (meth)acrylate, the patient avoids contact with any other (meth)acrylate [59, 61, 63, 68]. Although there are regulations for cosmetic products, there is still much to be done, especially in relation to nail polishes, as there is a lack of evidence-based literature to assist in the safety and choice of formulation components, since most of the available data on unwanted reactions are based on case reports [8, 61, 64, 68].

### 3.3. Main Adverse Effects Caused on Nails Related to the Use of (Meth)Acrylate Nail Polishes

Among the existing options for decorating nails, traditional nail polishes are the least harmful. In addition to the presence of (meth)acrylates, which are powerful sensitizers, the process of sanding gel nails and acrylic nails with milling machines causes wear on the nail plate and, consequently, damage to its structure. Gel polishes are usually removed with acetone, but one needs to be soaked in acetone for 10–20 min and covered with aluminum. Using acetone as a nail polish remover is also harmful to nails [27, 65, 71]. Cases of complaints related to thinning and fragility of nails after applying cosmetics based on (meth)acrylates have been reported. Chen et al. [72] confirmed the reduction in nail thickness after the application of gel polish by reflective confocal microscopy (RCM) and ultrasound. Both techniques showed thinning of the nail plate, with a 20% reduction measured by ultrasound and a reduction of almost 50% by RCM.

Borowczyk and Głowacki [73] confirmed changes in the concentration of cysteine and methionine caused by gel polishes and gel nails, which are the most important sulfur amino acids that build the structures of the nail plate. The study revealed that after 6 months of using this type of manicure, the average levels of sulfur amino acids in the studied samples were 22.1% lower in the case of cysteine and 36.5% lower in the case of methionine. This significant reduction in amino acids causes damage to the base of the nail plate, destroying the disulfide bonds. A reduction in nail thickness was also observed, from 0.50 ± 0.12 mm to 0.46 ± 0.12. This thinning of the nails is probably caused by the sanding step both to apply the product and to remove it.

Batory, Namiecinski, and Rotsztejn [74] evaluated the pH of nails in different situations: uncoated and coated. For uncoated nails, the average pH was 5.0 ± 0.5, while for nails coated with traditional nail polish, it was 5.8; for nails coated with gel nail polish, it was 6.77; for gel nails, it was 6.78; and for acrylic nails, the pH was 6.13. The value of 5.8 for nails coated with traditional nail polish is closer to the normal pH value of uncoated nails. Studies indicate that acidic pH is essential for keeping the microbiome regular. For pH values above 6.0, it can lead to infection and greater tendency to damage.

### 3.4. Other Problems Related to Nail Polish in General

It is also worth noting that exposure to certain harmful components is not always through direct contact with the nails and skin but rather through respiratory contact through contaminated air, mainly affecting nail professionals. These professionals are exposed daily to a series of chemicals, including volatile organic compounds (VOCs) that are part of the nail polish formulation [41, 49, 75, 76]. Many of these VOCs can cause skin, eye, and nose irritation, damage to the respiratory and reproductive systems, liver damage, and cancer. MMA was detected in 88% of the beauty salons analyzed, the highest concentration of which exceeds the reference concentration for chronic exposure through inhalation by more than 50 times [77].

Another issue to be debated is the disposal of these products. If there are few studies related to the toxicity of the components present in nail polish on human health, less is known about the impacts caused to the environment [78, 79]. Nail polishes are widely used around the world, and it is not known how many chemicals present in them are released into the environment. Felzenszwalb et al. [78] studied the negative effects of leached and solubilized extracts from nail polish, simulating the disposal of these products, and both extracts induced acute toxicity in zebrafish embryos, showing the risk to the aquatic ecosystem.

### 3.5. Traditional Nail Polish Beyond Esthetics

In addition to the esthetic appeal, the literature also reports the use of traditional nail polish for other purposes, since nails can absorb substances from the environment, as well as medications and drugs, through systemic circulation [74]. One of the main applications of nail polish has been as a form of treatment against fungal infections, since onychomycosis is a pathology that affects around 19% of the world's population and represents 50% of nail diseases [46, 80–83]. Treatment is through oral antifungals that require prolonged treatment and cause several systemic side effects, toxicity, and drug interactions [80, 84, 85]. On the other hand, formulations available for topical use are generally in the form of gels or creams and are not suitable for nails as they are easily removed by washing [80].

The advantage of traditional nail polish is its mechanical resistance and hydrophobicity. In this way, the study of the incorporation of medications into nail polish becomes an alternative to local treatment, avoiding the disadvantages of existing formulations and especially the disadvantages of systemic administration [80, 86]. The efficiency of using polymeric matrices in drug release occurs because the film creates a reservoir of the medication on the infected nail and the release occurs continuously [84, 86]. However, the keratinized structure of the nail acts as a barrier against the penetration of chemical agents, limiting the effectiveness of the nail polish. The nail barrier needs to be overcome for the drug to be delivered at the therapeutic dose necessary for the infection to be treated. One way to overcome this barrier is to use agents that act as a permeation enhancer, as they break the disulfide bonds present in the keratin structure [80, 87, 88].

Nail polish containing medication was first commercialized in 1992 under the name Loceryl, consisting of Eudragit RL 100, glycerol triacetate, butyl acetate, ethyl acetate, ethanol, and 5% amorolfine used as the active ingredients. In 1999, the FDA approved Penlac, composed of poly(methyl vinyl ether/maleic acid) butylmonoester, ethyl acetate, isopropyl alcohol, and 8% ciclopirox as active ingredients [89, 90]. Akhtar, Sharma, and Pathak [80] concluded in his review study that enamel-based antifungal formulations are stable and have the potential to be applied as a treatment for onychomycosis. Šveikauskaitè and Briedis [86] evaluated the effect of film-forming polymers on the release of naftifine hydrochloride to treat onychomycosis, and 98.5% of the drug was released within 6 h from the polymer matrix containing 15.0% Eudragit RL100.

Shah and Jobanputra [91] prepared a liposome-loaded nail polish formulation to increase the permeability of the drug terbinafine in the treatment of onychomycosis. Pandit et al. [88] prepared a transparent nail polish containing NC, ethylcellulose, ethyl acetate, dibutylphthalate, acetone, and salicylic acid as permeation enhancers and *Cissus quadrangularis* extract used as an antifungal. The nail polish showed good antifungal action against *Candida albicans,* and it was considered an effective antifungal medicine to treat onychomycosis. Machado et al. [83] added a quinoline derivative to traditional nail polish to be used as an antifungal agent, and the results were promising.

Souza et al. [81] incorporated Amphotericin B into a transparent nail polish to offer a topical treatment, considering that the nail polish can increase the local bioavailability of the medication. The obtained result confirmed the drug release in an adequate quantity to provide a local antifungal effect. Dantas et al. [82] synthesized silver nanoparticles (AgNPs) coated with humic acid (HA) to confer antifungal activity in samples of nail polish from Risqué. The obtained results indicated it as an effective alternative against onychomycosis, with HA improving the stability of the nanoparticles and its incorporation into nail polish did not influence its physical properties, remaining stable for 21 days.

Di Vito et al. [46] investigated the effectiveness of a modified nail polish formulation with seven essential oils with antimicrobial activity to treat fungal diseases such as onychomycosis. The Sally Hansen brand nail polish considered “16-free” was used as a treatment base, and the obtained result was satisfactory as a preventative and therapeutic. Nail polish has also been studied to provide calcium and silicon to the nails of diabetic patients to improve the damage caused by the disease. Calcium and silicon supplementation can help strengthen nails, and the development of topical treatments could be an attractive alternative, as the authors confirmed the delivery of elements to the nails through the nail polish [92].

An interesting study by Couteau, Paparis, and Coiffard [10] investigated the photoprotective effect of nail polish in cancer patients, whose disease treatment often results in nail-related side effects. The study assessed the effectiveness of 59 nail polishes against UV radiation and found that some had a sun protection factor (SPF) above 500. Brown and black were the most photoprotective, although it is not possible to predict the photoprotective effect based solely on their color. The study concluded that a dark-colored nail polish is not always more photoprotective than a light-colored nail polish.

A less common use, but which has been reported in the literature, is the possibility of using nail polish as forensic evidence. Just like samples of hair, blood, and other human fluids, nail polish remains can be important evidence in reconstituting a crime during a criminal investigation [9, 93–95]. Shimamoto, Terra, and Bueno [93] used the portable energy-dispersive X-ray fluorescence spectrometer (EDXRF) combined with chemometrics to analyze 42 samples of Brazilian nail polish from five different brands. The result obtained showed that the technique was able to discriminate and group the nail polish samples.

Chopi, Sharma, and Singh [94] used ATR-FTIR spectroscopy combined with chemometrics to differentiate 73 nail polish samples from different manufacturers and of similar shades. The results obtained were reproducible, and the discrimination of the samples was effective, but the authors state that more studies in the area are still needed. Khei et al. [95] used ATR-FTIR spectroscopy combined with chemometrics to discriminate 79 nail polish samples from eight brands and achieved high accuracy in predicting the analyzed samples.

## 4. Conclusion

Nail polishes are used around the world by thousands of people, and ensuring their safety for consumer's health is expected from regulatory agencies. However, even after 100 years of the modern nail polishes, there is no efficient inspection on its composition and their labels are not faithful to what is contained in the bottle. Even with the constant changes made by industries, some of the conventional nail polishes available on the market still contain some component considered toxic or sensitizing, while all nail cosmetics based on (meth)acrylates can be considered highly sensitizing and are responsible for numerous cases of ACD. Knowledge of the toxicity and health effects related to these components in nail polish formulations remains very limited, and the toxic effects observed in many studies do not follow a protocol, which makes it difficult to compare the results obtained and ends up dividing opinions. Inadequate labeling of nail polishes leads consumers who have some type of sensitivity to experience adverse reactions due to the lack of information about the presence of these allergens, even in formulations that state that they do not contain them. The exposure of users, as well as the professionals involved, also needs to be considered, since air is a route of contamination for the various volatile components present in these products. Therefore, the study of a formulation with biocompatible components becomes relevant to minimize risks to health and the environment.

## Figures and Tables

**Table 1:** Main ingredients used in nail polish over the years.

Solvents	Ethyl, n-butyl and propyl acetates, acetone, ethyl, n-butyl and isopropyl alcohols, diacetone alcohol, toluene, xylene, benzene, and heptane.

Film-forming polymers	Nitrocellulose, methacrylates, polyvinyl butyrate, cellulose acetate, ethyl cellulose, cellulose acetate propionate, cellulose acetate butyrate, and acrylate/styrene copolymer.

Resins	TSFR, tosylamide/epoxy resin, aryl-sulfonamide-formaldehyde, aryl-sulfonamide-epoxy resins, alkyd-type resins, polyester resins, polyurethane resins, acrylate/methacrylate copolymers, acrylate/styrene copolymer, phthalic anhydride/trimellitic anhydride/glycol copolymer (PA), adipic acid/neopentyl glycol/trimellitic anhydride copolymer (AA), phthalic anhydride/glycerin/glycydyl decanoate copolymer, polyvinyl butyral, trimethyl pentanyl diisobutyrate, acrylamide, dimethicone, sucrose acetate isobutyrate, and adipic acid/fumaric acid/phthalic acid/tricyclodecane dimethanol copolymer.

Plasticizers	DBP, DEP, dioctyl phthalate, triphenyl phosphate (TPHP), tricresyl phosphate, acetyl tributyl citrate (ATBC), camphor, trimethyl pentanediyl dibenzoate, trimethyl pentanyl diisobutyrate, ethyl tosylamide, diisobutyl adipate, glycerol carbonate, and dipropylene glycol dibenzoate.

Suspending agent	Stearalkonium bentonite and stearalkonium hectorite.

Pigments or dyes	CI 77491, CI 77499, CI 77891 (iron oxides), CI 77019 (mica), CI 77891 (titanium dioxide), CI 77002 (aluminum hydroxide), CI 77220 (calcium carbonate), CI 77510 (ferric ammonium ferrocyanide), CI 77163 (bismuth oxychloride), CI 77861 (tin oxide), CI 77120 (barium sulfate), CI 77288 (chromium oxide green), CI 77289 (chromium hydroxide green), CI 19140 (Acid Yellow 23 aluminum lake), CI 15850 (red 6 lake/red 7 lake), CI 17200 (Acid Red 33), CI 60725 (Violet 2), CI 60730 (Acid Violet 43), CI 15880 (Red 34 lake), CI 77007 (ultramarines), CI 42090 (Blue 1 lake), and CI 77266 (Black 2).

UV filters	Benzofenone-1, benzofenone-3, octocrylene, and drometrizole trisiloxane.

Others	Vitamins, calcium pantothenate, panthenol, calcium sodium borosilicate, citric acid, biotin, tocopherol, ferrous gluconate, silica, dimethicone, pentaerythrityl tetraisostearate, tocopheryl acetate, cysteine, calcium pantothenate, and dimethyl oxobenzo dioxasilane.

## Data Availability

Data sharing is not applicable to this article as no datasets were generated or analyzed during the current study.
